# Gain-of-Function of Stat5 Leads to Excessive Granulopoiesis and Lethal Extravasation of Granulocytes to the Lung

**DOI:** 10.1371/journal.pone.0060902

**Published:** 2013-04-02

**Authors:** Wan-chi Lin, Jeffrey W. Schmidt, Bradley A. Creamer, Aleata A. Triplett, Kay-Uwe Wagner

**Affiliations:** 1 Eppley Institute for Research in Cancer and Allied Diseases, University of Nebraska Medical Center, Omaha, Nebraska, United States of America; 2 Department of Pathology and Microbiology, University of Nebraska Medical Center, Omaha, Nebraska, United States of America; Emory University, United States of America

## Abstract

The Signal Transducer and Activator of Transcription 5 (Stat5) plays a significant role in normal hematopoiesis and a variety of hematopoietic malignancies. Deficiency in Stat5 causes impaired cytokine-mediated proliferation and survival of progenitors and their differentiated descendants along major hematopoietic lineages such as erythroid, lymphoid, and myeloid cells. Overexpression and persistent activation of Stat5 are sufficient for neoplastic transformation and development of multi-lineage leukemia in a transplant model. Little is known, however, whether a continuous activation of this signal transducer is essential for the maintenance of hematopoietic malignancies. To address this issue, we developed transgenic mice that express a hyperactive mutant of Stat5 in hematopoietic progenitors and derived lineages in a ligand-controlled manner. In contrast to the transplant model, expression of mutant Stat5 did not adversely affect normal hematopoiesis in the presence of endogenous wildtype *Stat5* alleles. However, the gain-of-function of this signal transducer in mice that carry *Stat5a/b* hypomorphic alleles resulted in abnormally high numbers of circulating granulocytes that caused severe airway obstruction. Downregulation of hyperactive Stat5 in diseased animals restored normal granulopoiesis, which also resulted in a swift clearance of granulocytes from the lung. Moreover, we demonstrate that Stat5 promotes the initiation and maintenance of severe granulophilia in a cell autonomous manner. The results of this study show that the gain-of-function of Stat5 causes excessive granulopoiesis and prolonged survival of granulocytes in circulation. Collectively, our findings underline the critical importance of Stat5 in maintaining a normal balance between myeloid and lymphoid cells during hematopoiesis, and we provide direct evidence for a function of Stat5 in granulophilia–associated pulmonary dysfunction.

## Introduction

Signal Transducers and Activators of Transcription 5 (Stat5a and Stat5b) mediate extracellular signals from a variety of cytokine receptors and are therefore essential for the growth and differentiation of many cell types including those of hematopoietic lineages. Mice deficient in either Stat5a or Stat5b show defects in the prolactin-induced functional differentiation of the mammary gland [1] or in sexual dimorphism in the control of body size mediated by growth hormone [2]. The phenotypic examination of hypomorphic mutant mice that express low levels of truncated Stat5a and Stat5b (*Stat5^ΔN^* ^/*ΔN*^) revealed that both Stat5 isoforms have redundant functions. *Stat5^ΔN^* ^/*ΔN*^ double mutant mice exhibit abnormalities during erythropoiesis and reduced proliferation of peripheral T cells [3]–[5]. The Cre-mediated ablation of the entire *Stat5* locus from the murine genome caused much more severe phenotypes and resulted in perinatal lethality due to anemia and other defects [6]. Subsequent studies using Stat5a/Stat5b conditional knockout mice also showed that the combined functions of these evolutionarily conserved transcription factors are critical for the homeostasis and differentiation of hematopoietic stem cells and derived descendants along the lymphoid lineage [7]–[11]. Moreover, Stat5 is required for granulocyte macrophage colony-stimulating factor receptor (GM-CSF) signaling and controls granulopoiesis by promoting the generation of granulocytes from granulocyte-macrophage progenitors (GMPs) as well as the survival of mature neutrophils [12], [13].

The phenotypes associated with a knockout of Stat5 in mice provided guidance to the identification of the first germline mutations in the coding region of the *STAT5B* gene in patients who were insensitive to growth hormone (GH) and who did not carry any mutations in the GH receptor [14]–[16]. Interestingly, the majority of STAT5B deficient cases in humans were associated with symptoms of severe infection, autoimmune diathesis, and lymphocytic interstitial pneumonitis. These patients also exhibited a reduction in the numbers of regulatory T cells, suggesting that loss of STAT5B in humans appears to be sufficient for the initiation of certain immune phenotypes as well as chronic lung disease [17].

Both STAT5 isoforms are frequently overexpressed and activated in a broad range of human cancers and hematologic malignancies. Cytokine-independent cell growth and survival, which is a hallmark of neoplastic transformation, can be caused by aberrant autocrine signaling as well as genetic and epigenetic changes in intracellular signal networks that involve tyrosine kinases and negative regulators [18]. Chromosomal translocations that lead to the formation of hyper-active JAK2 fusion proteins such as TEL-JAK2, BCR-JAK2, and PCM1-JAK2 signal through STAT5 and are frequently detected in various leukemia subtypes [for references see reviews by Valentino and Pierre (2006) and Ghoreschi et al. (2009) [19], [20]. Additionally, missense mutations in the *JAK2* gene (e.g. JAK2V617F) have been shown to be associated with many myeloproliferative disorders [21]–[24]. Besides JAK2, STAT5 can be persistently activated in leukemias by the BCR/ABL tyrosine kinase and mutations in c-KIT or FLT-3 [25]–[28]. Using retroviral gene transfer of a hyperactive mutant of Stat5 (caStat5a-S710F) into hematopoietic stem/progenitor cells and their transplantation into recipient mice, Moriggl and colleagues [29] demonstrated that a persistent activation of this signal transducer was sufficient to cause multi-lineage leukemia in mice. Then again, expression of the same mutant under regulation of the lymphoid-specific Eµ enhancer was weakly oncogenic, and only 6 out of 50 transgenic mice developed B-cell malignancies after a long latency [30]. This suggests that secondary mutations are required for disease onset in this transgenic line. Regardless of whether active Stat5 acts as a weak or strong oncogene depending on the experimental model, it still remains to be elucidated whether a Stat5-induced hematopoietic malignancy requires a continuous expression of the mutant signal transducer for disease maintenance.

In this report, we describe the development and analysis of transgenic lines that express the S710F mutant of Stat5a in hematopoietic progenitors and derived lineages in a ligand-controlled manner to assess whether a sustained activation of Stat5 is required for the growth and survival of preneoplastic or neoplastic hematopoietic cells following disease initiation. We show here that, depending on the levels of endogenous Stat5, the persistent activation of this signal transducer causes excessive granulopoiesis and prolonged survival of granulocytes in circulation. Upon extravasation, these cells can cause severe pulmonary obstruction and lethality. Stat5 is clearly important for maintaining a normal balance between lymphoid and myeloid cells during hematopoiesis, and all of these major phenotypes are reversible through downregulation of hyperactive Stat5.

## Results

### Generation of transgenic mice that express hyperactive Stat5 in a temporally and spatially controlled manner

**Figure 1:**
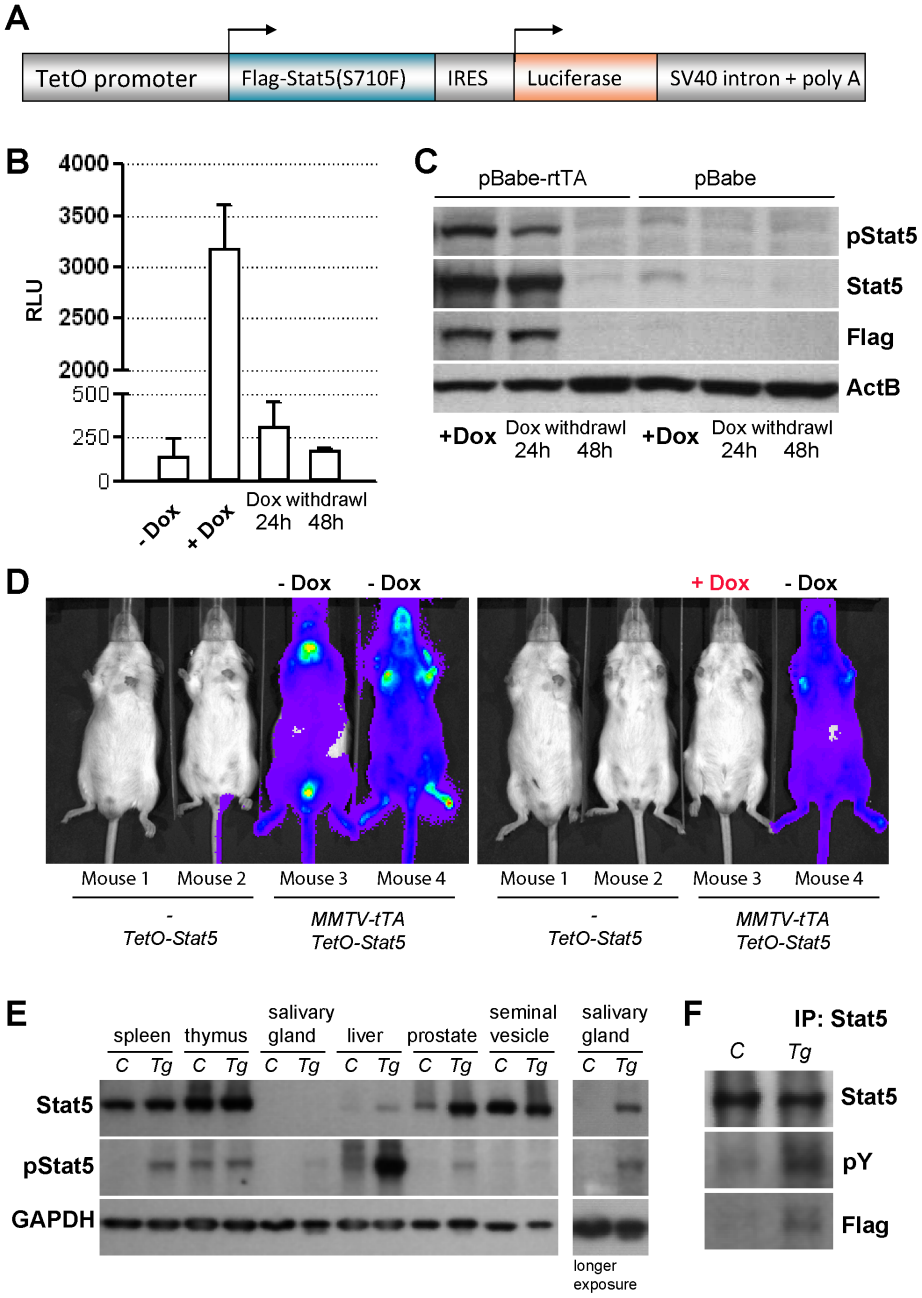
Generation and analysis of transgenic mice that co-express a Flag-tagged hyperactive mutant of Stat5a (S710F) and luciferase under control of the tetracycline-controlled operator (TetO). A. Schematic outline of the transgene. B. Conventional luciferase assay on mouse embryonic fibroblasts (MEFs) derived from TetO-Stat5 mice that were infected with a pBabe-rtTA-puro retroviral construct and maintained in the absence or presence of doxycycline (± Dox). C. Western blot analysis to verify the expression of Flag-tagged, exogenous Stat5 (tyrosine phosphorylated and total) in MEFs derived from TetO-Stat5 mice that were infected with the pBabe-rtTA-puro or pBabe-puro control vector. Cells were maintained in the presence of Dox, and the withdrawal of this ligand led to a significant decline in transgenic Stat5 expression within 48 hours in cells expressing the reverse transactivator protein (rtTA). D. In vivo bioluminescence imaging of two MMTV-tTA TetO-Stat5 double transgenic mice (mouse 3 and 4) and their TetO-Stat5 single transgenic controls (mouse 1 and 2) prior to administration of Dox (left panel). The right panel shows the same animals after mouse 3 was treated for 48 hours with Dox. E. Western blot to assess the expression of total and tyrosine phosphorylated Stat5 in a panel of tissues from double transgenic mice (Tg) and their TetO-Stat5 single transgenic controls (C). F. IP/Western blot for total and active Stat5 on spleen tissues of a control mouse (C) and a double transgenic animal expressing exogenous Stat5a (Tg).

We developed transgenic mice in which the expression of exogenous Stat5 can be targeted to hematopoietic stem cells and derived lineages in a ligand regulatable manner. Upon expression of a tetracycline-controlled transactivator, the TetO-Stat5 responder transgene (Fig. 1A) permits the co-expression of Stat5 and a luciferase reporter for *in vivo* bioluminescence imaging. To facilitate a gain-of-function of this signal transducer, we utilized a Flag-tagged S710F mutant of Stat5a, which has been reported to exhibit a persistent tyrosine phosphorylation in the absence of cytokines [29]. Following the generation of 15 founders by pronuclear injection, the correct temporally controlled expression of the TetO-Stat5 transgene was examined in detail in two independent founder lines (11651 and 11676). For this purpose, we derived primary mouse embryonic fibroblasts from both lines and infected them with a retrovirus expressing the reverse transactivator (pBabe-rtTA, Tet-ON). Expression of luciferase and Stat5 was only detected when these cells were treated with doxycycline (Dox), and the levels declined within 48 hours following the withdrawal of the ligand (Fig. 1B and 1C). To target the expression of hyperactive Stat5 to hematopoietic cells, we crossed TetO-Stat5 mice with a transgenic strain that expresses the tetracycline-controlled transactivator (tTA, Tet-OFF) under control of the MMTV-LTR [31]. In contrast to the rtTA (Tet-ON), which we used in the cell culture experiments (Fig. 1B and 1C), the tTA (Tet-OFF) transactivator is better suited for a constitutive expression of TetO-driven transgenes *in vivo* for a prolonged period since a continuous administration of Dox is not required. Besides expression in various secretory organs, the MMTV-tTA construct is active in hematopoietic cells and has been successfully used in the past to establish models of B-cell leukemia and lymphoma [32]–[34]. Expression of the luciferase reporter was only observed in MMTV-tTA TetO-Stat5 double transgenic animals using *in vivo* imaging, and the TetO-Stat5 transgene alone does not exhibit any background activation in the absence of the transactivator (Fig. 1D, left panel). Upon administration of Dox, the tTA-mediated transactivation and expression of the TetO-Stat5 construct can be rapidly and completely silenced (Fig. 1D, right panel). The immunoblot analysis revealed that the MMTV-tTA-mediated transactivation of the TetO-Stat5 transgene occurred in multiple secretory organs, and overexpression of exogenous Stat5 was readily detectable in the salivary gland and in the prostate (Fig. 1E). In organs that express high levels of endogenous Stat5 (spleen, thymus, and seminal vesicle), the transgenic protein was proportionally less abundant. However, we observed a significant increase in the steady-state levels of active Stat5 in splenocytes, which was confirmed using immunoprecipitation (IP) and western blotting (Fig. 1F). In summary, while the expression of exogenous Stat5 did not lead to a significant elevation in total Stat5, there was a noticeable increase in the activation of Stat5 in hematopoietic cells.

**Figure 2:**
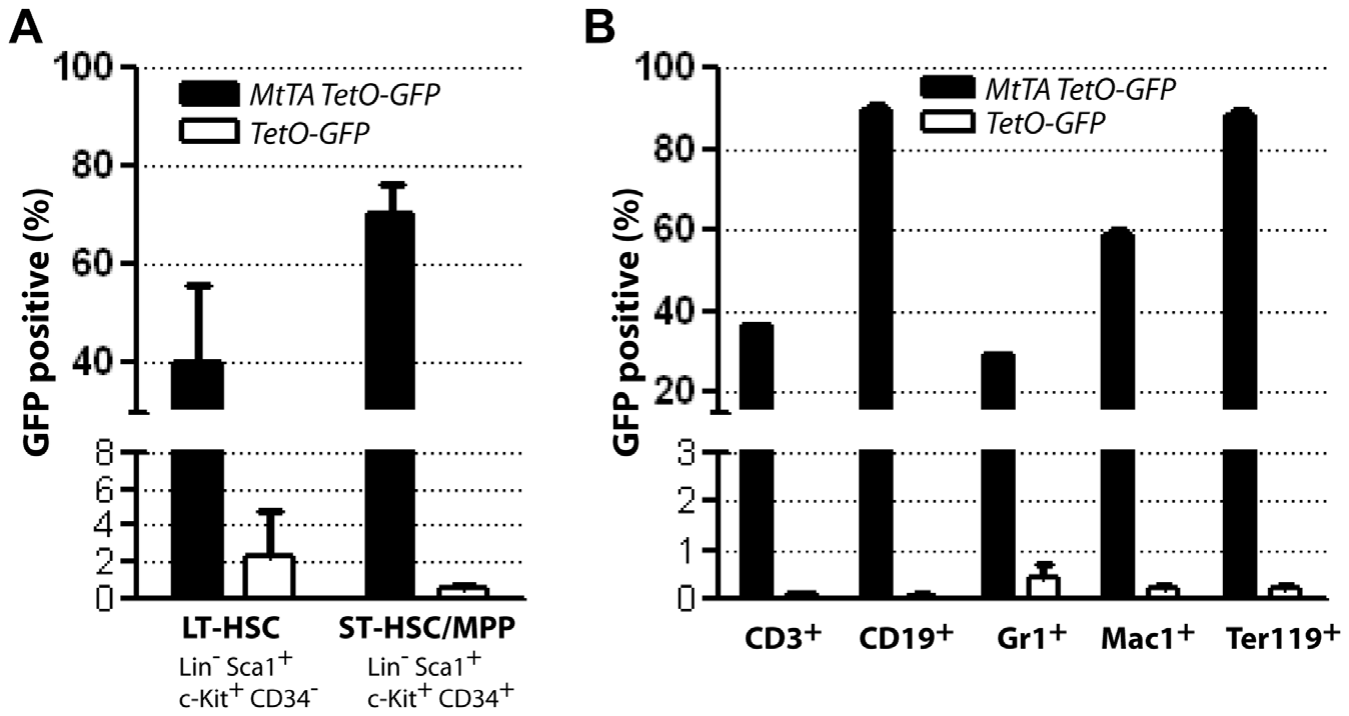
MMTV-tTA-mediated transactivation of TetO-responder transgenes in hematopoietic stem cells and derived lineages. Flow cytometric analysis of the presence of the GFP reporter in long-term and short-term (LT-HSC, ST-HSC) hematopoietic stem cells (**A.**) as well as lymphoid, myeloid, and erythroid cells (**B.**) in the bone marrow of MMTV-tTA TetO-GFP double transgenic mice and their TetO-GFP single transgenic controls.

To assess whether the MMTV-tTA-mediated expression of TetO-driven responder genes is restricted to lymphoid cells, we examined in more detail the expression profile of the MMTV-tTA transgene in major hematopoietic cell types. For this purpose, we generated MMTV-tTA TetO-H2B-GFP double transgenic mice and performed a flow cytometric analysis for GFP expression in bone marrow cells that were labeled with various markers for long-term (LT-HSC) and short-term (ST-HSC) hematopoietic stem cells (Fig. 2A; Fig. S1) as well as various lineage-specific markers (Fig. 2B; Fig. S2). This study revealed that, in addition to lymphoid cells, the MMTV-tTA is active in a significant subset of hematopoietic stem cells (HSCs) under nonselective conditions (e.g., without expression of an oncogene). Within differentiated hematopoietic lineages, the tTA is expressed in erythroid and B cells and to a lesser extend in T cells and granulocytes.

**Figure 3:**
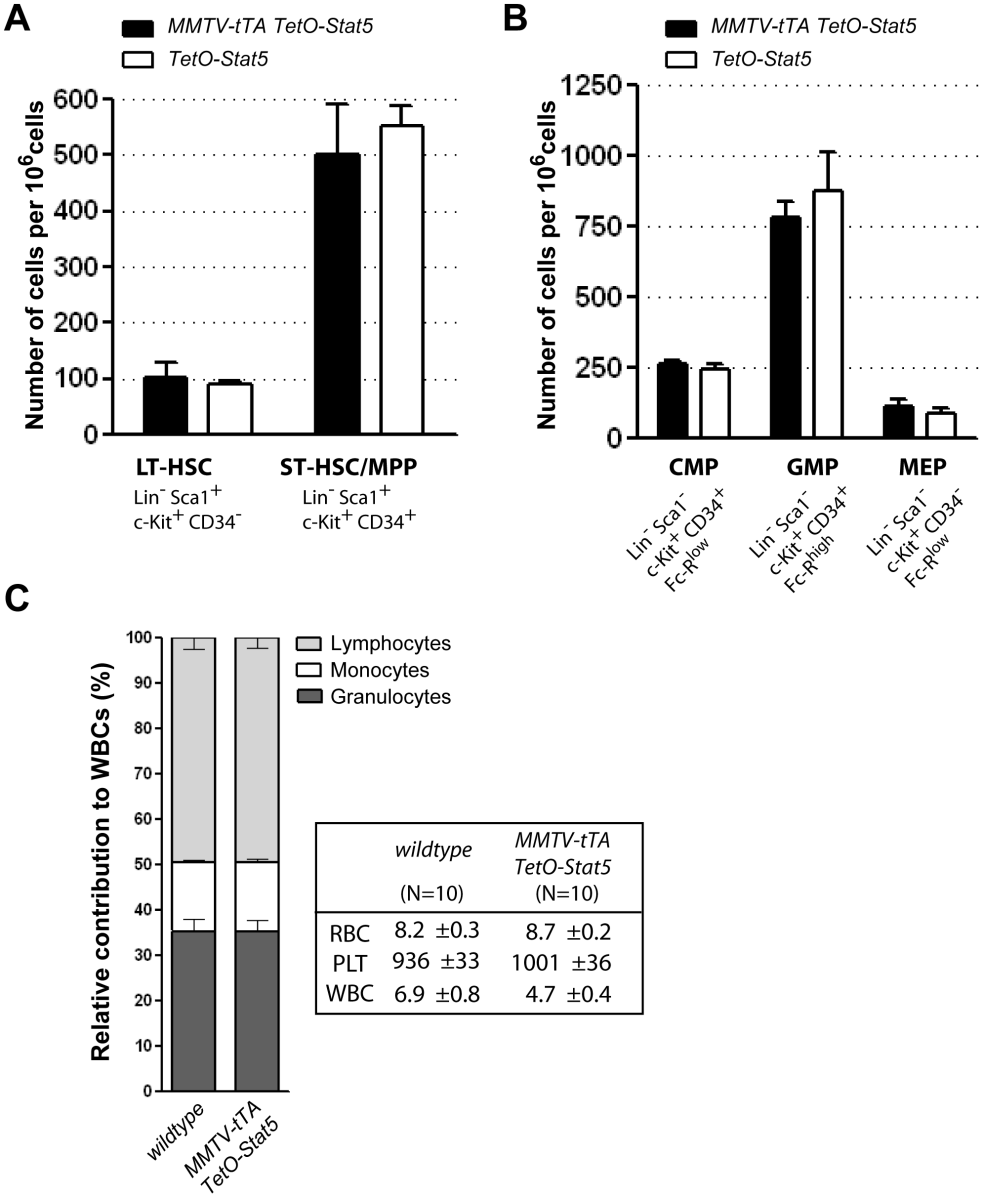
Mice expressing hyperactive Stat5 under control of the MMTV-tTA have normal blood cell counts. Relative numbers of long-term and short-term (LT-HSC, ST-HSC) hematopoietic stem cells (**A.**) as well as common myeloid (CMP), granulocyte-macrophage (GMP), and megakaryocyte-erythrocyte (MEP) progenitors (**B.**) in the bone marrow of MMTV-tTA TetO-Stat5 double transgenic mice and their TetO-Stat5 single transgenic controls. **C.** Absolute numbers of peripheral blood cells (10^3^/mm^3^) and relative contribution of lymphocytes, monocytes, and granulocytes to the total number of white blood cells. RBC, red blood cells; PLT, platelets; WBC, white blood cells.

Next, we assessed possible effects caused by the expression of hyperactive Stat5 on the number of hematopoietic stem and progenitor cells. The flow cytometric analysis revealed that gain-of-function of Stat5 did not significantly change the number of particular stem/progenitor cells in the bone marrow (Fig. 3A, 3B, and Figures S3 and S4). In addition, the MMTV-tTA TetO-Stat5 double transgenic animals exhibited blood counts that were similar to single transgenic controls (Fig. 3C). Based on the previous report that expression of Stat5 from a retroviral vector leads to a rapid onset of multi-lineage leukemias [29], we were surprised to note that MMTV-tTA TetO-Stat5 double transgenic mice did not develop any hematopoietic malignancies within 18 months of age.

### Expression of hyperactive Stat5 in mutant mice with Stat5a/b hypomorphic alleles leads to a fatal increase in circulating granulocytes that infiltrate the lung

**Figure 4:**
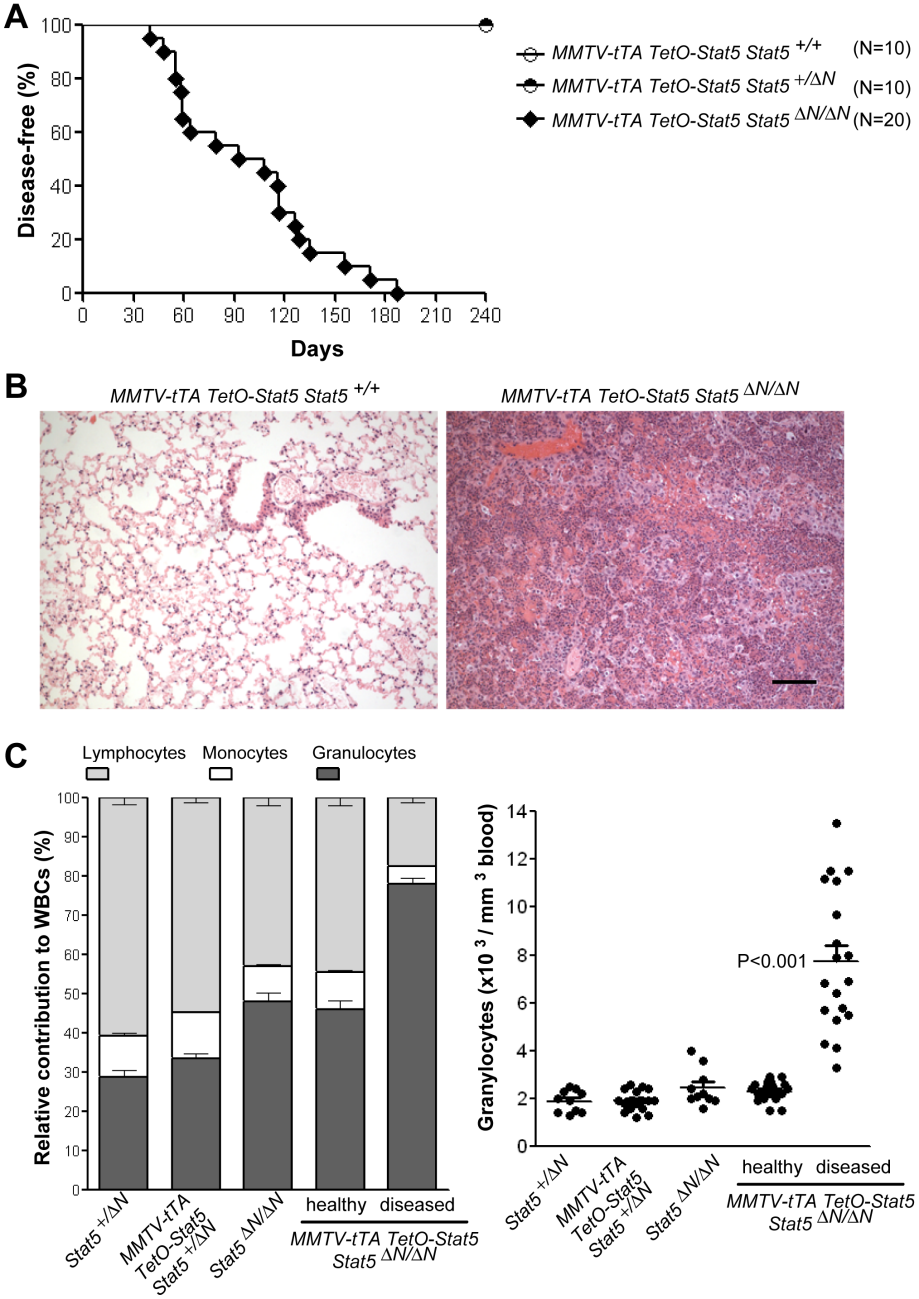
Gain-of-function of Stat5a in mice with hypomorphic *Stat5a/b* alleles causes a fatal increase in circulating granulocytes. A. Kaplan–Meier curve illustrating the disease-free survival of mice expressing hyperactive Stat5 (MMTV-tTA TetO-Stat5) in the presence of two wildtype endogenous *Stat5a/b* alleles (*Stat5^+/+^*) or in animals that carry *Stat5a/b* hypomorphic alleles in a heterozygous or homozygous configuration (*Stat5^+/^* ^Δ*N*^ and *Stat5* ^Δ*N/*Δ*N*^). B. Sections of lung tissues from diseased MMTV-tTA TetO-Stat5 *Stat5* ^Δ*N/*Δ*N*^ mice and healthy MMTV-tTA TetO-Stat5 controls. Bar represents 100 µm. C. Relative contribution of lymphocytes, monocytes, and granulocytes to the total number of white blood cells (WBCs) (left panel) and total granulocyte counts (right panel) in healthy and diseased MMTV-tTA TetO-Stat5 *Stat5* ^Δ*N/*Δ*N*^ mice and controls (*P* value, *t* test).

Recent evidence suggests that a disease phenotype induced by a hyperactive Jak2/Stat5 signaling pathway is influenced by the level of signaling and the ratio of mutant to wildtype protein [35], [36]. Since the level of exogenous Stat5 under regulation of the MMTV-tTA is not substantially higher than the endogenous Stat5, we next examined whether the loss of endogenous Stat5 might lead to differences in the functionality of the exogenous mutant protein. For this purpose, we generated mice that carry the MMTV-tTA and TetO-Stat5 transgenes in a Stat5 hypomorphic mutant background (*Stat5* ^Δ*N*/Δ*N*^). Homozygous *Stat5* ^Δ*N*/Δ*N*^ mice are known to express very low levels of an N-terminal truncated Stat5 protein. Overexpression of transgenic, hyperactive Stat5 in the Stat5 hypomorphic background had a profound impact on the disease-free lifespan of these animals compared to their controls (Fig. 4A). Starting at two months of age, a significant subset of MMTV-tTA TetO-Stat5 *Stat5* ^Δ*N*/Δ*N*^ mice started to exhibit signs of severe airway obstruction and had to be euthanized. The histopathological analysis revealed a substantial extravasation of immune cells into the lung (Fig. 4B). We also observed hematopoietic cells, albeit to a much lesser extent, in the liver and kidney (not shown). Initially, young animals exhibited a granulocyte count that was similar to controls. However, prior to or during disease onset, we observed a sharp increase in the relative number of granulocytes and a significant elevation in the total number of granulocytes in circulation (Fig. 4C). Hence, although the MMTV-tTA is only expressed in approximately one third of all Gr1-positive cells in comparison to 90% of erythroid and B-cells (Fig. 2B), the gain-of-function of Stat5 lead to a selective elevation in the absolute number of granulocytes in circulation whereas the number of all other white and red blood cells as well as platelets remained unaltered.

### Downregulation of Stat5 swiftly restores normal granulopoiesis and reverts extravasation of granulocytes to the lung

**Figure 5:**
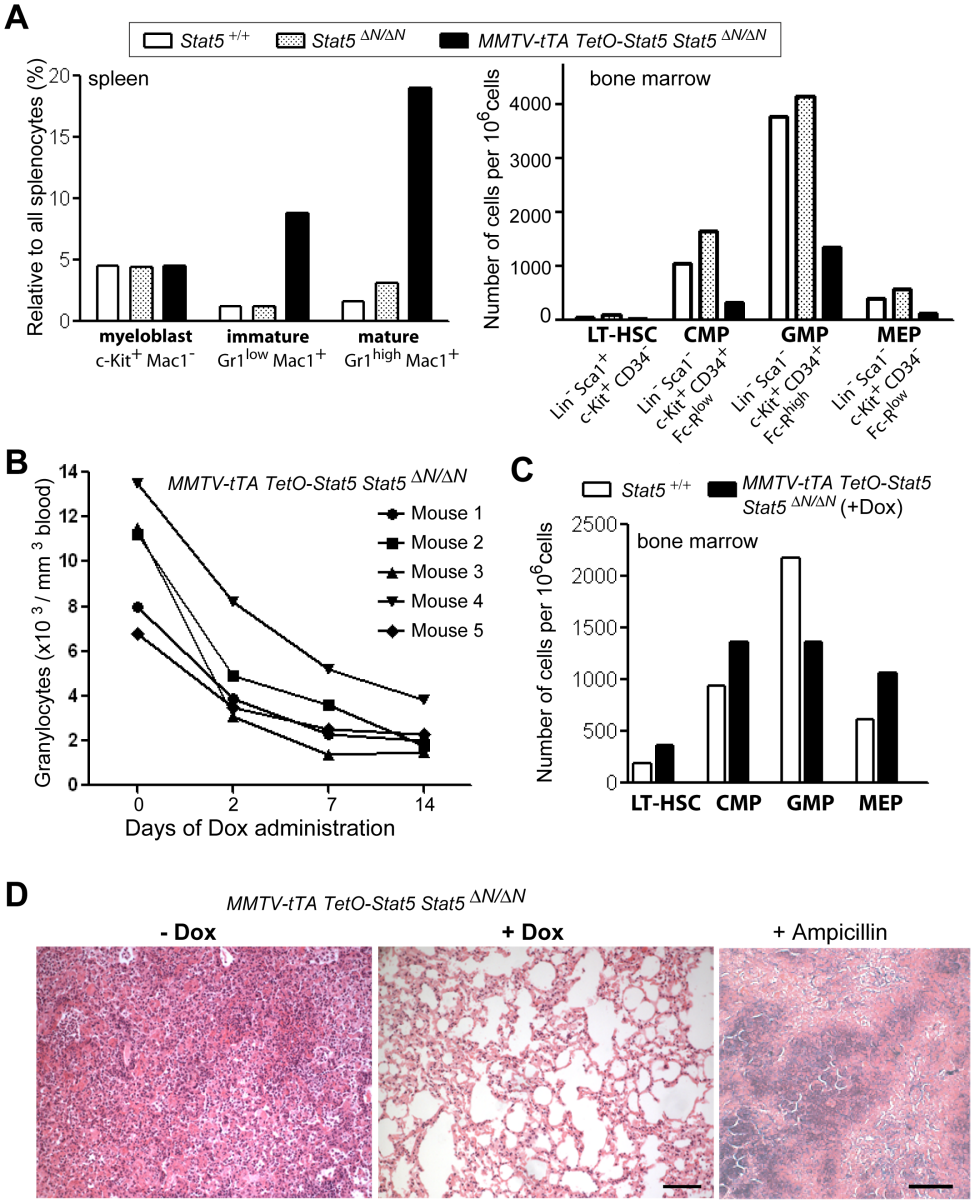
Downregulation of exogenous Stat5a leads to clearance of excessive granulocytes in circulation and in the lung as well as restoration of a normal progenitor pool. A. Relative contribution of myeloblasts and immature and mature granulocytes among the total number of splenocytes (left panel) and numbers of long-term hematopoietic stem cells (LT-HSC) as well as common myeloid (CMP), granulocyte-macrophage (GMP), and megakaryocyte-erythrocyte (MEP) progenitors in the bone marrows of diseased MMTV-tTA TetO-Stat5 *Stat5* ^Δ*N/*Δ*N*^ mice and their controls. B. Changes in the number of granulocytes in five diseased animals that were treated for 14 days with Dox. C. Numbers of hematopoietic stem cells and progenitors in a wildtype control and a diseased MMTV-tTA TetO-Stat5 *Stat5* ^Δ*N/*Δ*N*^ mouse following Dox administration. D. Sections of lung tissues from diseased MMTV-tTA TetO-Stat5 *Stat5* ^Δ*N/*Δ*N*^ mice prior to and following downregulation of Stat5 (±Dox). The right panel shows the lung of a mouse treated with ampicillin to control for nonspecific effects of Dox as an antibiotic as opposed to its direct control of Stat5 expression. Bars represent 100 µm (left, middle) and 200 µm (right).

The flow cytometric analysis of splenocytes from diseased animals and their controls showed that the gain-of-function of Stat5 resulted in a moderate increase in immature and a substantial elevation in the number of mature granulocytes (Fig. 5A, left panel and Fig. S5A). In contrast, the relative amount of myeloblasts remained unchanged. While hypomorphic Stat5-deficient mice with or without exogenous Stat5 have comparable numbers in long-term hematopoietic stem cells, expression of hyperactive Stat5 led to a reduction in common myeloid progenitors (CMPs) as well as a decline in granulocyte/monocyte (GMP) and megakaryocyte/erythroid (MEP) progenitors in the bone marrow (Fig. 5A, right panel and Fig. S5B).

To assess whether the survival of circulating granulocytes, including those that extravasated into the lung, depended on the continuous expression of hyperactive Stat5, we treated MMTV-tTA TetO-Stat5 *Stat5* ^Δ*N/*Δ*N*^ mice with Dox after they developed high granulocyte counts and exhibited signs of airway obstruction. Within only two to seven days, the administration of Dox led to a significant decline in the number of circulating granulocytes (Fig. 5B), and after 2 weeks of treatment, the granulocyte count was normalized to the level of healthy control mice (compare to Fig. 4C). Notably, the downregulation of exogenous Stat5 also resulted in the restoration of the drastically depleted pool of CMPs and MEPs in the bone marrow (Fig. 5C and Fig. S6). Following treatment with Dox, the mice were also visibly healthier, and the histological analysis of the lungs revealed that, unlike the untreated controls, the administration of Dox resulted in a complete clearance of granulocytes from the airway system (Fig. 5D). Since treatment with another antibiotic (i.e., ampicillin) did not alleviate the severity of this condition, the reversal of the phenotype was likely a specific result of the downregulation of exogenous Stat5 and not a broader effect of the antibiotic properties of doxycycline. Animals receiving Dox remained disease free during a prolonged treatment period. However, the withdrawal of this ligand led to a recurrence of elevated granulocyte counts within two weeks (not shown). This suggests that the downregulation of hyperactive Stat5 did not cause a selective elimination of precursors that are the cellular basis for accelerated disease recurrence upon re-expression of active Stat5.

### Hyperactive Stat5 promotes excessive granulopoiesis in a cell autonomous manner

**Figure 6:**
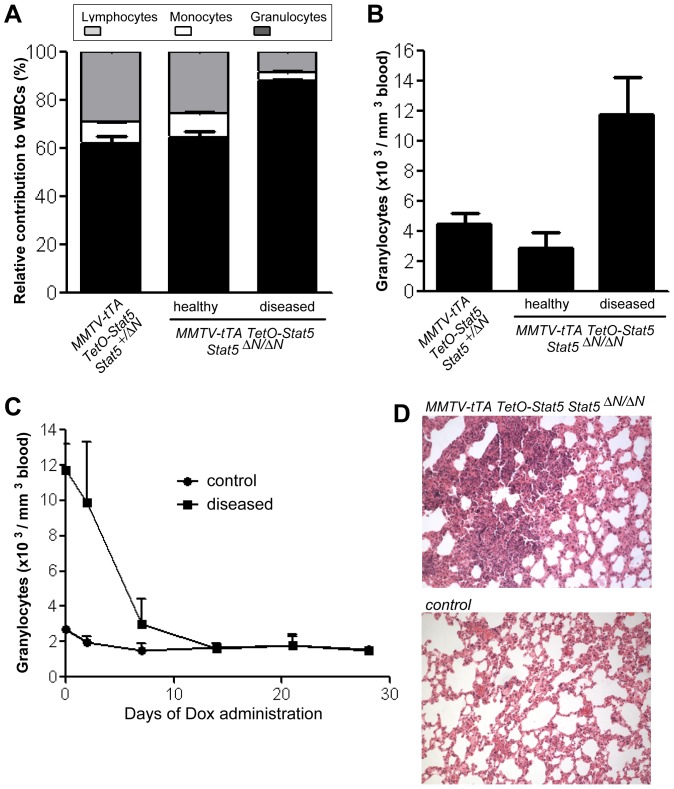
Excessive granulopoiesis is controlled by hyperactive Stat5 in a cell autonomous manner. **A**. Relative contribution of lymphocytes, monocytes, and granulocytes to the total number of white blood cells (WBCs) and **B.** total granulocyte counts in healthy and diseased recipients that were engrafted with bone marrow cells from MMTV-tTA TetO-Stat5 *Stat5* ^Δ*N/*Δ*N*^ mice and MMTV-tTA TetO-Stat5 *Stat5^+/ΔN^* controls. **C.** Changes in the number of granulocytes in three diseased recipient mice and controls that were treated for 28 days with Dox. **D.** Corresponding sections of lung tissues from diseased recipient mice and their normal controls.

It has been reported that Stat5 hypomorphic mutant mice (*Stat5* ^Δ*N/*Δ*N*^) mice develop mild neutrophilia through a cell extrinsic mechanism, i.e. an overproduction of granulocyte colony-stimulating factor in liver endothelial cells [37] While the relative amount of granulocytes among WBCs was increased on average by 10–15%, only two of these mutant animals actually exhibited a marginal increase in the total number of granulocytes (Fig. 4C, right panel). Severe granulophilia was only observed in Stat5 hypomorphic mutant mice when they also express hyperactive Stat5. To assess whether the disease onset in these mice was due to the proposed extrinsic production of cytokines or a Stat5-mediated intrinsic mechanism, we transplanted bone marrow cells from MMTV-tTA TetO-Stat5 *Stat5* ^Δ*N/*Δ*N*^ mice as well as MMTV-tTA TetO-Stat5 *Stat5* ^Δ*N/wt*^ controls into conditioned wildtype mice. Recipient animals expressing hyperactive Stat5 in the hypomorphic mutant background developed high relative and absolute granulocyte counts that were similar to donor mice (Fig. 6A and 6B). This suggested that the excessive granulopoiesis was likely a direct effect of the gain-of-function of Stat5 within myeloid progenitors and their differentiated descendants. This cell intrinsic mechanism does not require an increased production of G-CSF in extra-hematopoietic cell types. This assumption is supported by the fact that the treatment of diseased animals with Dox resulted in the clearance of circulating granulocytes and restoration of normal granulopoiesis that is indistinguishable from control animals (Fig. 6C). Although wildtype recipient mice expressing mutant Stat5 exhibited some abnormalities in their lungs (Fig. 6D), the extravasation of granulocytes to the airway system was less widespread compared to the donors.

**Figure 7:**
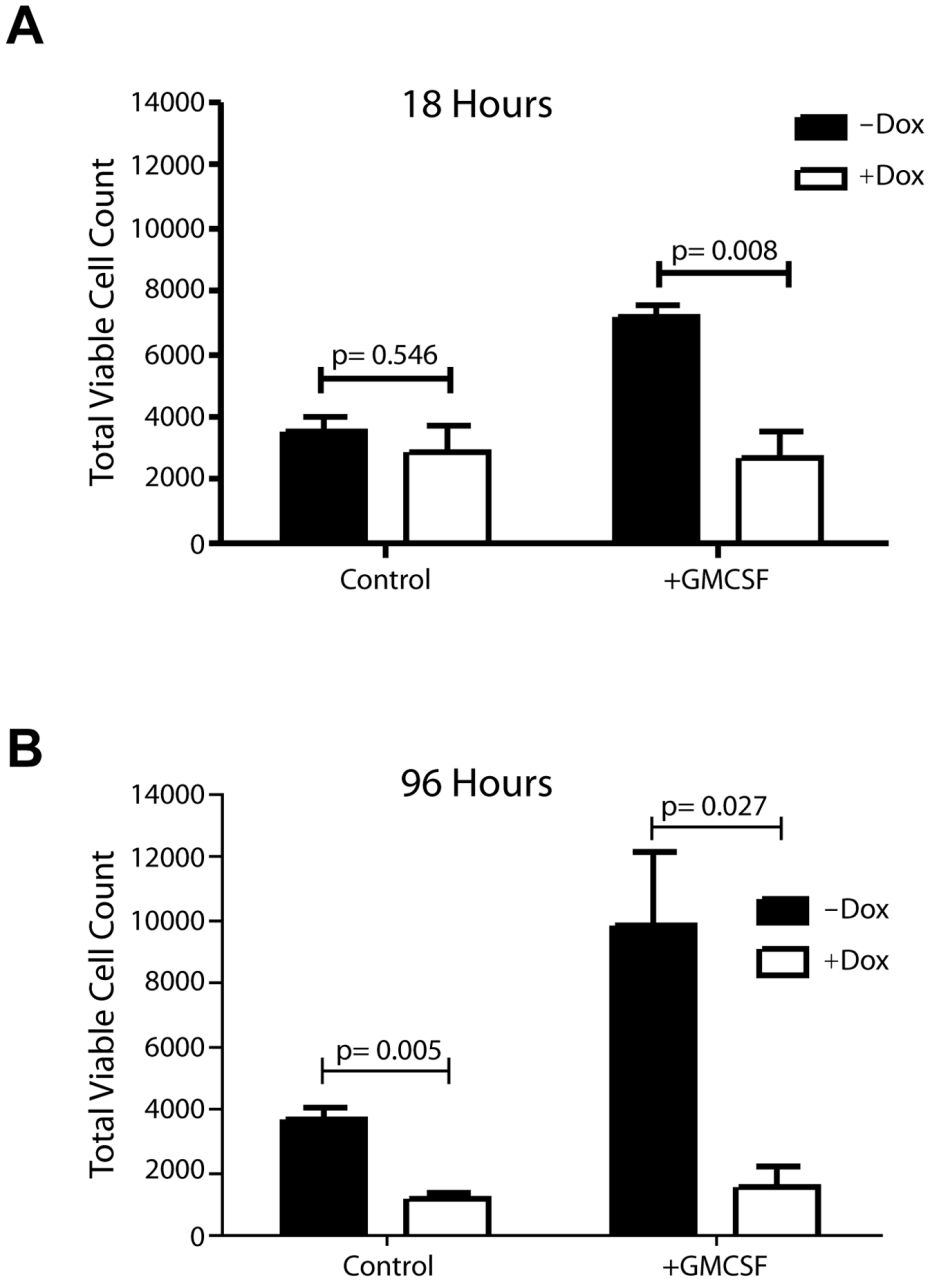
Gain-of-function of Stat5 promotes survival of circulating granulocytes ex vivo. Viable cell counts of peripheral WBCs that consisted of more than 90% granulocytes from diseased MMTV-tTA TetO-Stat5 *Stat5* ^Δ*N/*Δ*N*^ mice that were maintained in culture for 18 (**A.**) or 96 hours (**B.**) in the presence and absence of Dox and GM-CSF (*P* value, *t* test).

**Figure 8:**
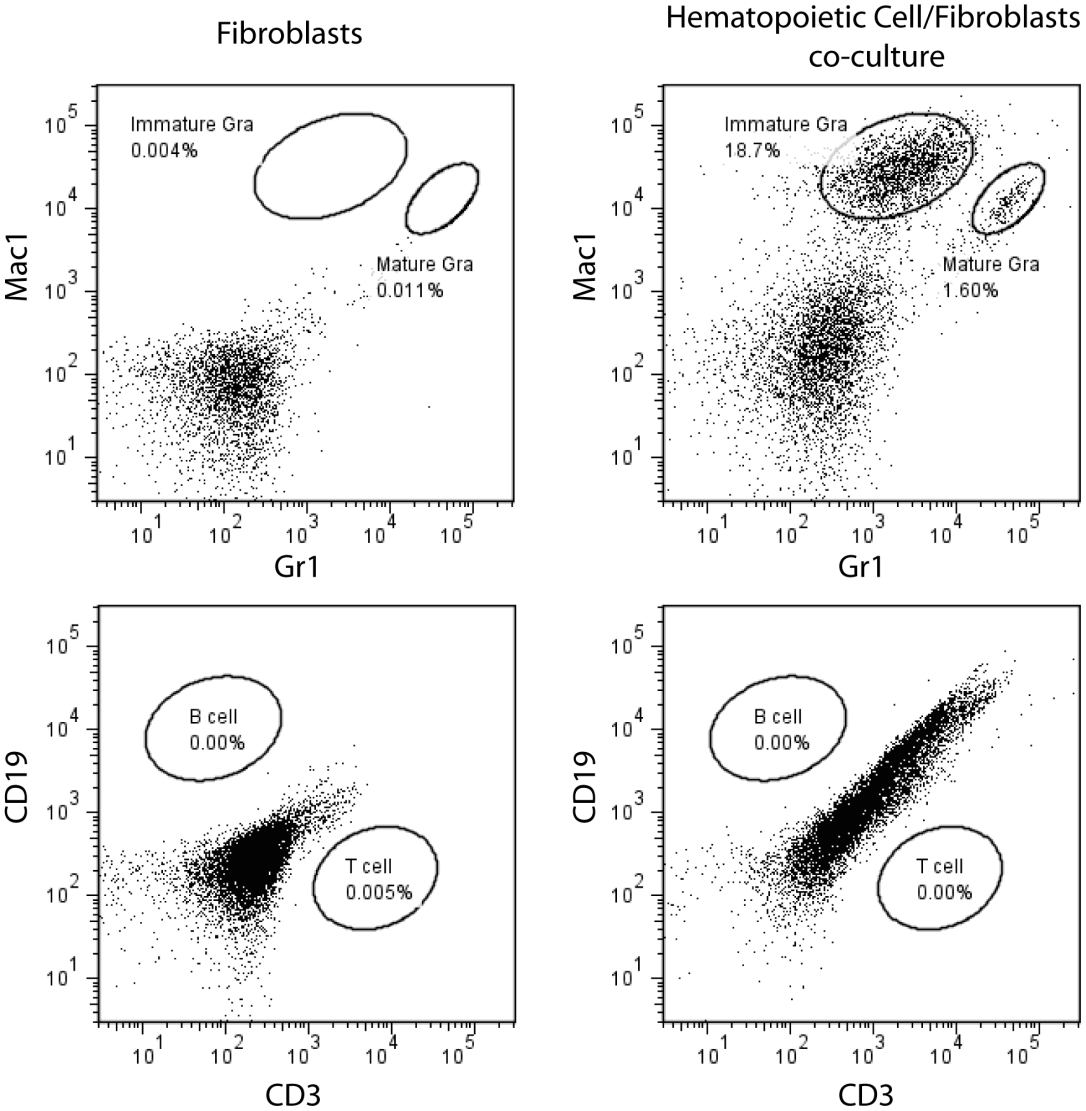
The majority of lung-derived hematopoietic cells expressing exogenous Stat5 that can be maintained for a prolonged period in culture are immature granulocytes. Flow cytometric analysis of various lineage markers on lung-derived hematopoietic cells that were maintained *ex vivo* together with pulmonary fibroblasts for 12 weeks.

**Figure 9:**
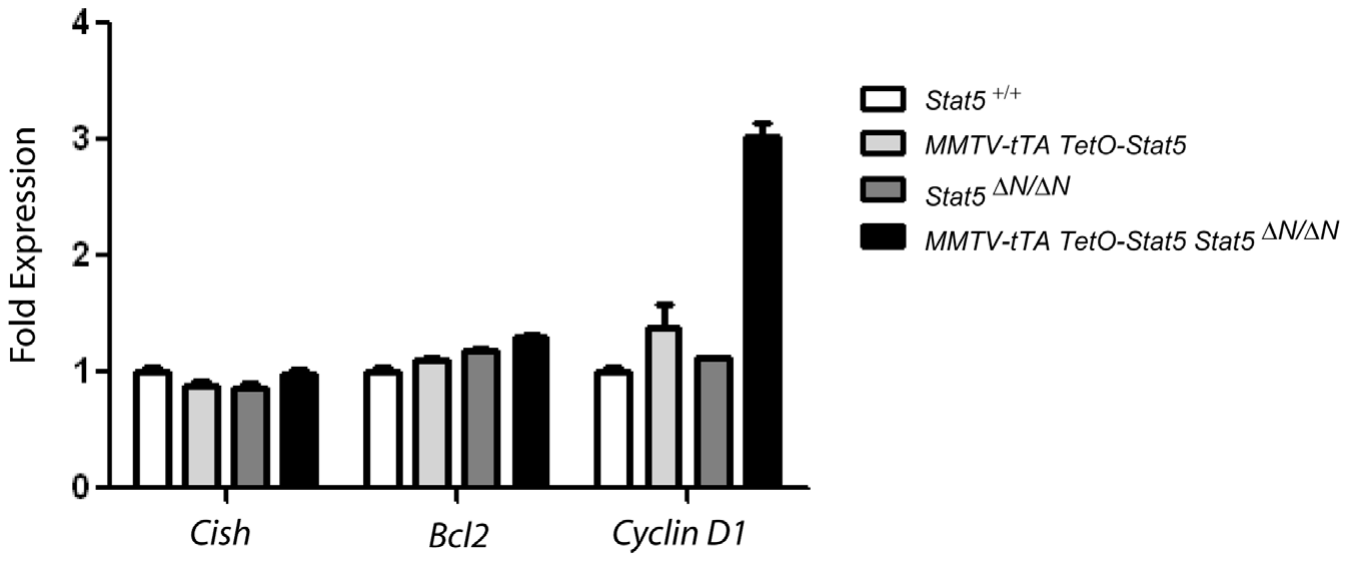
Hematopoietic cells from mice with a gain-of-function of Stat5 show a significant increase in the transcriptional activation of *Cyclin D1*. Quantitative real-time RT-PCR to assess the transcriptional activation of *Cyclin D1*, *Bcl2*, and *Cish* in bone marrow derived hematopoietic cells of experimental mice that express hyperactive Stat5 in the absence or presence of *Stat5a/b* hypomorphic alleles (MMTV-tTA TetO-Stat5 *Stat5^+/+^* and MMTV-tTA TetO-Stat5 *Stat5* ^Δ*N/*Δ*N*^). Wildtype littermates (*Stat5^+/+^* and Stat5 hypomorphic mutants (*Stat5* ^Δ*N/*Δ*N*^) served as controls.

It has been reported previously that Stat5 is essential for GM-CSF signaling, and Stat5a/b conditional double knockout mice show decreased numbers of mature neutrophils [12]. Since we observed significantly elevated numbers of circulating granulocytes that also accumulated over time in the lungs of our Stat5 overexpression model, we assessed next whether the gain-of-function of Stat5 promoted a prolonged survival in addition to the increased production and release of these particular hematopoietic cells into circulation. For this purpose, we isolated peripheral white blood cells (WBCs) through retro-orbital blood draw from diseased MMTV-tTA TetO-Stat5 *Stat5* ^Δ*N/*Δ*N*^ mice. These WBCs had a relative contribution of more than 90% granulocytes and were maintained *ex vivo* for up to 96 hours in the presence and absence of Dox and GM-CSF (Fig. 7). After 96 hours in culture, less than 10% of seeded cells lacking Stat5 (<2,000 of 20,000 plated cells per well) were still viable (Fig. 7 B, open bars). Even when treated with GM-CSF, these cells cannot be effectively maintained *in vitro.* In contrast, more than twice the amount of cells that express Stat5 were alive during that period, and we observed a clear synergistic effect of Stat5 expression and growth factor treatment, which yielded a significant increase in cell survival (Fig. 7 B, solid bars). In a separate experiment, we isolated hematopoietic cells from the lung, and we were able to maintain them on top of lung-derived fibroblasts for at least 12 weeks. This suggested that many extravasated cells were alive and a subset may have acquired an immortal state *in vivo* or after a short term in culture. The flow cytometric analysis revealed that the majority of these proliferating cells were immature granulocytes, and some cells underwent differentiation into mature granulocytes in culture (Fig. 8). The notion that myeloid precursors expressing hyperactive Stat5 show accelerated proliferation and an increase in survival might be supported by the fact that mice expressing exogenous Stat5 exhibited a significant increase in the activation of the *Cyclin D1* gene, i.e. a direct transcriptional target of Stat5 in hematopoietic cells [38], and elevated levels of *Bcl2* (Fig. 9).

## Discussion

As a signal transducer and transcription factor downstream of many cytokines, Stat5 acts as a mitogenic factor in most cell types, including hematopoietic progenitors. It has been recently suggested that activation of Stat5 maintains the quiescence of hematopoietic stem cells (HSCs) during steady-state hematopoiesis [39]. The conditional deletion of both Stat5 isoforms in HSCs using Mx1-Cre in adult mice led to increased stem cell cycling and depletion of the long-term HSC pool. We did not observe any significant changes in the numbers of LT-HSCs and ST-HSCs in MMTV-tTA TetO-Stat5 transgenic mice. In part, this might be the result of a mosaic expression pattern of the MMTV-tTA, which appears to be active in only 40% of LT-HSCs as opposed to 70% of ST-HSCs in this transgenic line. Despite expression of hyperactive Stat5 in over 90% of erythroid and B cells as well as the majority of macrophages, MMTV-tTA TetO-Stat5 transgenic mice did not develop multi-lineage leukemia similar to the retroviral gene transfer and transplantation model generated by Moriggl and colleagues [29]. We initially assumed that the transactivation of the TetO-Stat5 transgene by the MMTV-tTA might not have been efficient enough, and we, therefore, crossed both TetO-Stat5 responder lines with Eμ-tTA and Tal1-tTA transgenics that have been utilized to generate multiple leukemia and lymphoma models [40], [41]. None of the resulting double transgenics developed hematopoietic malignancies within 12 months of age. We also used retroviral and lentiviral gene transfer to express the tTA in HSCs and differentiated lineages in bone marrow cells derived from TetO-Stat5 transgenics to induce a cytokine storm that is generally associated with the transplantation of infected cells into conditioned wildtype recipient mice. Despite verification of the permanent engraftment of the infected bone marrow cells, recipient animals did not develop leukemia or lymphoma after a prolonged latency. Additionally, we examined a possible effect of the genetic background on Stat5-induced neoplastic transformation. For this purpose, we transferred the MMTV-tTA and one of the TetO-Stat5 transgenes (line 11676) into the C57/Bl6 strain through eight or more backcrosses, but none of the double transgenic mice developed hematopoietic abnormalities within 12 months of age. The results of our combined efforts to generate a reversible, Stat5-induced leukemia model indicate that this signal transducer appears to be a rather weak oncogene that requires additional, cancer-initiating mutations to trigger a malignant phenotype. Hence, our observations are similar to Eμ-Stat5 mice that exhibit a very low penetrance in cancer formation [30]. This is perhaps not unique to the hematopoietic system. Evidently, the expression of wildtype or constitutively active Stat5 in the mammary epithelium is only weakly oncogenic [42]–[44]. Moreover, despite expression of mutant Stat5 in other secretory tissues such as the prostate and salivary gland in our model (see Fig. 1E), we also did not see any consistent malignant changes in these organs in aging MMTV-tTA TetO-Stat5 transgenics.

While expression of mutant Stat5 in the presence of the wildtype, endogenous protein did not seem to cause any major phenotypic abnormalities, the transfer of the MMTV-tTA and TetO-Stat5 transgenes into the Stat5 hypomorphic mutant background (*Stat5* ^Δ*N/*Δ*N*^) had a profound effect on the differentiation of hematopoietic cells and the wellbeing of the mice. The results of our study indicate that expression of hyperactive Stat5a in the presence of hypomorphic *Stat5a/b* alleles promotes the numeric expansion of immature and mature granulocytes and a synchronous depletion of progenitors (i.e., CMPs and MEPs). In addition, the gain-of-function of Stat5 leads to an increase in the survival of peripheral granulocytes and their lethal extravasation to multiple organs, most prominently, the lung. All of these major phenotypes are reversible through downregulation of the mutant Stat5. Our observations confirm previous reports that highlight the importance of Stat5 in maintaining a normal balance between lymphoid and myeloid cells during hematopoiesis as well as essential functions of this transcription factor downstream of GM-CSF signaling [7], [12], [13], [37]. It might be possible that the low-level expression of N-terminally truncated, endogenous Stat5 plays some role in the initiation of the phenotypes. We confirmed that *Stat5* ^Δ*N/*Δ*N*^ mice have a slight increase in the relative number of granulocytes. This was suggested to be a consequence of emergency granulopoiesis that might overcompensate for the intrinsic survival defect in this hematopoietic lineage [37]. Expression of hyperactive Stat5 might act in a synergistic manner and delay the turnover of circulating granulocytes and thereby promote the severity of the phenotype. But this process might not depend exclusively on the elevated secretion of G-CSF from liver endothelial cells in *Stat5* ^Δ*N/*Δ*N*^ mutants as previously proposed [37]. The transplant experiments in this study suggest that hyperactive Stat5 is still able to promote excessive granulopoiesis in a cell autonomous manner. This notion is supported by the reversibility of the severe consequences of Stat5 overexpression in the MMTV-tTA TetO-Stat5 *Stat5* ^Δ*N/*Δ*N*^ transgenics and their derived hematopoietic transplants in wildtype recipients.

The increase in granulocyte count and extravasation into the lung were typically observed after two to six months. This might imply that additional genetic or epigenetic changes were required to promote disease manifestation. In contrast to the initiation of the disease, the reactivation of the transgene expressing mutant Stat5 following complete disease remission led to a re-accumulation of granulocytes in circulation after just 14 days. This suggests that precursors, which are highly responsive to the expression of mutant Stat5 and that might carry secondary hits, can survive the downregulation of the transgene. The subsequent reactivation of exogenous Stat5 then leads to an accelerated expansion of immature and mature granulocytes. The implication of this observation is that Stat5 needs to be effectively and permanently inactivated to prevent rapid disease recurrence.

Despite clear evidence that exogenous Stat5 promotes extended survival of peripheral granulocytes, the underlying cause for their extravasation into the lung remains unknown. Since the lungs of diseased mice resemble necrotizing pneumonitis on the histopathological level, we initially assumed the presence of foreign organisms as disease initiating agents. A gram, Ziehl–Neelsen, and silver staining was performed and evaluated by Dr. Robert Cardiff (UC Davis), and all tests were negative, suggesting that various types of bacteria, pseudomonas, legionella, and fungi like Pneumocystis and Candida did not seem to play a significant role in disease manifestation. At this point, however, we cannot exclude that the extravasation is trigged by a variety of environmental toxins. In addition to pathogens, we considered that a deregulated expression of Stat5 in the lung epithelium might fuel a local production of cytokines that provoke excessive extravasation. However, a quantitative RT-PCR assay to assess the expression of various cytokines did not reveal any significant differences among disease-free and diseased animals with or without expression of exogenous Stat5. Regardless of the underlying events that trigger the massive accumulation of immune cells in the lungs of mice expressing hyperactive Stat5a in a Stat5a/b deficient background, our observations might potentially have clinical implications in humans. As mentioned in the *Introduction* section, chronic lung disease and immune dysfunction appear to be common among patients that are deficient in STAT5B [17]. It is very likely that the remaining STAT5A plays a significant role in this process. Unfortunately, the expression and activation of STAT5A has not been specifically assessed in these patients, but this particular STAT5 isoform appears to be the predominant target for GM-CSF signaling [45]. More importantly, STAT5 has been shown to be an essential mediator for GM-CSF signaling and survival of lung-derived granulocytes in a veterinary animal model for asthma [46]. If lack of STAT5B results in a hyperactivation of the remaining STAT5A in the myeloid lineage, targeting JAK2/STAT5 locally might alleviate some of the severe symptoms in STAT5B deficient patients for whom there are currently limited treatment options available to improve the complex immune defects.

## Materials and Methods

### Generation of TetO-Stat5 transgenic mice

The pTet-Splice vector (kindly provided by Dr. Jun-Lin Guan, Cornell University) was modified by inserting of a short DNA oligo into the *Xho*1 site upstream of the TetO-promoter sequence to introduce additional *Swa*I/*Not*I restriction sites. The Flag-tagged mutant of *Stat5a* (S710F), which is a gift from Dr. Richard Moriggl (LBI-CR, Vienna), was inserted into the *Sma*I site in front of an IRES-Luciferase construct. The entire Stat5-IRES-Luc cassette was cloned as a blunted *Not*I/*Sac*I fragment into the *Eco*RV site of the modified pTet-Splice vector. The transgene was released from the vector backbone using *Not*I, gel purified, and injected into FVB zygotes by the UNMC Mouse Genome Engineering Core Facility. Fifteen transgenic founder lines were obtained following pronuclear injection, and two lines (11651 and 11676) were used for experiments described here. The Mouse Genome Informatics (MGI) nomenclature for both of these strains is Tg(tetO-Stat5a*S710F,-luc)11651Kuw and Tg(tetO-Stat5a*S710F,-luc)11676Kuw. Offspring were screened by genomic PCR with primers recognizing the junction (approximately 345 bps) between the TetO/CMVmin promoter and the *Stat5a* cDNA (primer 1978: 5′-CCG TCA GAT CGC CTG GAG ACG-3′; primer 1977: 5′-GCC TGA ATC CAG CCC GCC ATG-3′).

### Other genetically modified mouse strains

MMTV-tTA [129/B6.Cg-Tg(MMTVtTA)1Mam] transgenic mice [31] were kindly provided by Dr. Lothar Hennighausen (NIH). In addition to the experiments described here, we recently performed a more detailed analysis of the expression profile of this strain in extrahematopoietic tissues [47]. TetO-H2B-GFP [Tg(tetO-HIST1H2BJ/GFP)47Efu/J] transgenic mice [48] were obtained from the Jackson Laboratory. Genetically modified mice that carry both targeted alleles for *Stat5a* and *Stat5b* [Stat5a^tm1Jni^/Stat5b^tm2Jni^] on chromosome 11 [3], designated here as *Stat5* ^Δ*N/*Δ*N*^, were kindly provided by Dr. James N. Ihle (St. Jude Children's Research Hospital). The MMTV-tTA transgene and *Stat5a/b* targeted alleles were transferred into an FVB background (n = 8). This study was carried out in strict accordance with the recommendations in the Guide for the Care and Use of Laboratory Animals of the National Institutes of Health. The protocol was approved by the Institutional Animal Care and Use Committee of the University of Nebraska Medical Center (IACUC#: 06-082-03).

### Administration of Dox and in vivo imaging of luciferase expression

Complete repression of the TetO-Stat5-IRES-Luc transgene was achieved through administration of freshly prepared doxycycline (Dox; Sigma, St. Louis, MO) in the drinking water (2 mg/ml supplemented with 50 mg/ml sucrose). The expression and activity of the luciferase reporter gene was monitored using *in vivo* bioluminescence imaging machine (IVIS200, Caliper Life Sciences, Alameda, CA) as described previously [49], [50]. According to the manufacture's recommendations, luciferin (1 mg D-luciferin potassium salt in 0.21ml 1× PBS) was injected intraperitoneally ten minutes prior to the imaging procedure. The mice were kept under anesthesia (isoflurane) during the acquisition of the images that were collected at intervals ranging from ten seconds to four minutes.

### Immunoprecipitation and western blot analysis

The preparation of whole-cell extracts of clarified cell lysates and tissues homogenates as well as the experimental procedures for immunoprecipitation (IP) and western blot analysis were described in detail elsewhere [51]. The following antibodies were used: anti-Stat5 antibodies E289 (Abcam, Cambridge, MA) and N-20 as well as anti-β-actin (I-19) (Santa Cruz Biotechnology, Santa Cruz, CA); anti-pStat5 antibody (Cell Signaling Technology, Danvers, MA); anti-Flag antibody M2 (Sigma, St. Louis, MO); anti-pY antibody 4G10 (Millipore, Billerica, MA); and anti-GAPDH antibody D16H11 (Cell Signaling Technology, Danvers, MA).

### Histology

Lung tissues from healthy and diseased mice were fixed overnight at 4°C in buffered formalin, repeatedly washed in 1× PBS, and paraffin embedded. Histological sections were prepared and stained with hematoxylin and eosin (H&E) at the UNMC Tissue Sciences Core Facility. Images of histological slides were taken on a Zeiss AxioImager microscope (Carl Zeiss, Inc., Germany) equipped with a SPOT FLEX camera (Diagnostic Instruments, Inc., Sterling Heights, MI).

### Peripheral blood cell counts

Approximately 50 µl of blood was collected from the retro-orbital sinus of mice into a tube containing EDTA using capillary tubes. The blood cell counts were measured on a Scil Vet ABC hematology analyzer (scil animal care company Ltd., Gurnee, IL).

### Flow cytometry analysis

orbital venous of anesthetized mice. Bone marrow cells were flushed from tibias and femurs. The cells of spleen or thymus tissues were squeezed through a 70 µm cell strainer (Becton Dickinson, Franklin Lakes, NJ) with the plunger of a syringe. Red blood cells were removed from these samples using the erythrocyte lysing kit WBL1000 VitaLyse ® from BioE (St. Paul, MN) according to the manufacturer's protocol. The remaining nucleated cells were then washed once with 1× PBS, resuspended in 1× PBS/1% FBS and stained with saturating amounts of antibodies against cell surface markers of various hematopoietic cells. For the cell linage analysis, the following antibodies were used: CD3-Alexa 647, CD19-Percp-Cy5.5, Ly6G (Gr-1)-APC-Cy7, CD11b (Mac-1)-Alexa 700, and Ter119-PE-Cy7. All the antibodies were purchased from BD Biosciences or eBioscience. For the analysis of stem or progenitor cells, the bone marrow cells were first purified by lineage depletion, using a magnetically labeled cocktail of biotinylated antibodies against lineage antigens as well as anti-biotin microbeads and a magnetic separator (Miltenyi Biotec, Auburn, CA). Subsequently, cells were labeled with APC-conjugated streptavidin (Invitrogen, Molecular Probe, Eugene, OR), and stained with Ly-6A/E (Sca-1)-Percp-Cy5.5, CD117 (c-kit)-PE-Cy7 as well as additional antibodies to define hematopoietic stem cells and progenitors as described [52]–[55]. The flow cytometry was performed on an LSRII and analyzed with the DIVA software (Becton Dickinson, Franklin Lakes, NJ).

### Transplantation of bone marrow cells into wildtype mice

Bone marrow cells were harvested from the femurs and tibias of transgenic mice and resuspended in 1× PBS. 5× 10^6^ cells per 0.2 ml were injected into the tail veins of sublethally irradiated (1,300 rads) FVB recipient mice. Mice were housed in microisolator cages under sterile conditions.

### Ex vivo cell cultures and survival assay

Peripheral white blood cells (WBCs) were isolated through retro-orbital blood draw from diseased transgenic mice, and erythrocytes were subsequently lysed using the VitaLyse® kit. Purified WBCs were comprised of 95% granulocytes according to hematological analysis (Scil Vet ABC hematology analyzer). 20,000 viable WBCs were plated in triplicates into 96-well plates, and cells were maintained in RPMI 1640 medium in the presence or absence of 40 ng/ml GM-CSF (PeproTech, Rocky Hill, NJ) and/or 10 µg/ml doxycycline. Cell viability was then determined after 18 and 96 hours using the trypan blue exclusion assay.

### Quantitative real-time RT-PCR

Total RNA was extracted from bone marrow cells using standard guanidinium thiocyanate-phenol-chloroform extraction. A Superscript II kit from Invitrogen with Oligo(dT) primers was utilized to perform the fist strand synthesis. Quantification of *Bcl2*, *Cyclin D1*, *Cish* and *Gapdh* expression was performed using iQ SYBR green Supermix (Bio-Rad) and gene-specific primer sets (*Bcl2*: 5′-CTC GTC GCT ACC GTC GTG ACT TCG-3′ and 5′-CAG ATG CCG GTT CAG GTA CTC AGT C-3′; *Cyclin D1*: 5′- CAG ACG GCC GCG CCA TGG AA-3′ and 5′- AGG AAG TTG TTG GGG CTG CC-3′; *Cish*: 5′- GCA GAG AAT GAA CCG AAG GTG C-3′ and 5′- GGA AGC TAG AAT CGG CGT ACT C-3′; *Gapdh*: 5′- GTG TCC GTC GTG GAT CTG ACG-3′ and 5′- CAA CCT GGT CCT CAG TGT AGC-3′). The quantitative PCRs (qPCRs) were carried out in triplicate in a CFX96 real-time PCR detection system (Bio-Rad, Hercules, CA). The expression values obtained were normalized against *Gapdh*, and standard curves were generated from the same samples.

### Statistical analysis

All graphic illustrations and statistics were performed with Prism 5 software (GraphPad Software, Inc., La Jolla, CA). Data are expressed as mean ±SD unless otherwise indicated and were compared using an unpaired Student *t* test. A *P* of less than .05 was considered significant.

## Supporting Information

Figure S1 **Representative flow cytometric blots showing the presence of GFP in hematopoietic stem cells of MMTV-tTA TetO-GFP double transgenic mice and their TetO-GFP single transgenic controls.** These blots correspond to the bar graph shown in Fig. 2A.
(PDF)

Figure S2 **Representative flow cytometric blots showing the presence of GFP in differentiated hematopoietic lineages of MMTV-tTA TetO-GFP double transgenic mice and their TetO-GFP single transgenic controls.** These blots correspond to the bar graph shown in Fig. 2B.
(PDF)

Figure S3 **Representative flow cytometric blots of the relative numbers of long-term and short-term (LT-HSC, ST-HSC) hematopoietic stem cells in the bone marrow of MMTV-tTA TetO-Stat5 double transgenic mice and their TetO-Stat5 single transgenic controls.** These blots correspond to the quantitative analysis shown in Fig. 3A.
(PDF)

Figure S4 **Representative flow cytometric blots of common myeloid (CMP), granulocyte-macrophage (GMP), and megakaryocyte-erythrocyte (MEP) progenitors in the bone marrow of MMTV-tTA TetO-Stat5 double transgenic mice and their TetO-Stat5 single transgenic controls.** These blots correspond to the quantitative analysis shown in Fig. 3B.
(PDF)

Figure S5 **Representative flow cytometric blots of the contribution of myeloblasts and immature and mature granulocytes among the total number of splenocytes (A) and numbers of long-term hematopoietic stem cells (LT-HSC) as well as common myeloid (CMP), granulocyte-macrophage (GMP), and megakaryocyte-erythrocyte (MEP) progenitors (B) in the bone marrows of diseased MMTV-tTA TetO-Stat5** ***Stat5*** **^Δ^** ^***N*/Δ*N***^ **mice and their controls.** The blots shown in panel A correspond to the bar graph illustrated in the left panel of Fig 5A. The blots from the backgating shown in panel B correspond to the bar graph shown in the right panel of Fig 5A.
(PDF)

Figure S6 **Representative flow cytometric blots of long-term hematopoietic stem cells (LT-HSC) as well as common myeloid (CMP), granulocyte-macrophage (GMP), and megakaryocyte-erythrocyte (MEP) progenitors in the bone marrows of a wildtype control and a diseased MMTV-tTA TetO-Stat5** ***Stat5*** **^Δ^** ^***N*/Δ*N***^ **mouse following Dox administration.** These backgated blots against the total number of bone marrow cells correspond to the bar graph shown in Fig 5C.
(PDF)
