# Supplementary figures and images for: Gain-of-Function of Stat5 Leads to Excessive Granulopoiesis and Lethal Extravasation of Granulocytes to the Lung
Source: PLoS One. 2013 Apr 2;8(4):e60902. doi: 10.1371/journal.pone.0060902 (PMC3614894; doi:10.1371/journal.pone.0060902)

**Figure S1**

Lin and Schmidt et al.

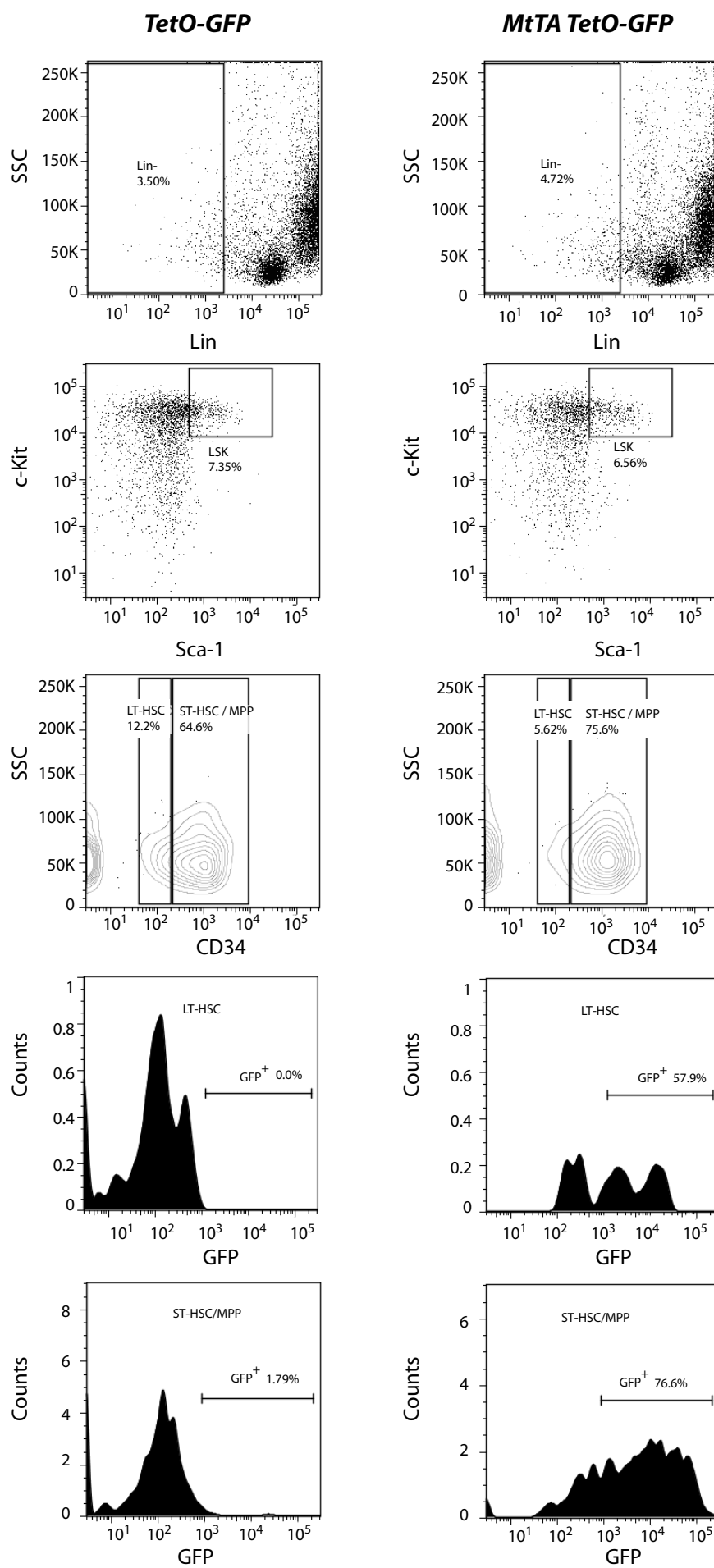

Supplement: Figure S1 — Representative flow cytometric blots showing the presence of GFP in hematopoietic stem cells of MMTV-tTA TetO-GFP double transgenic mice and their TetO-GFP single transgenic controls. These blots correspond to the bar graph shown in Fig. 2A. (PDF) [file pone.0060902.s001.pdf]

**Figure S2**

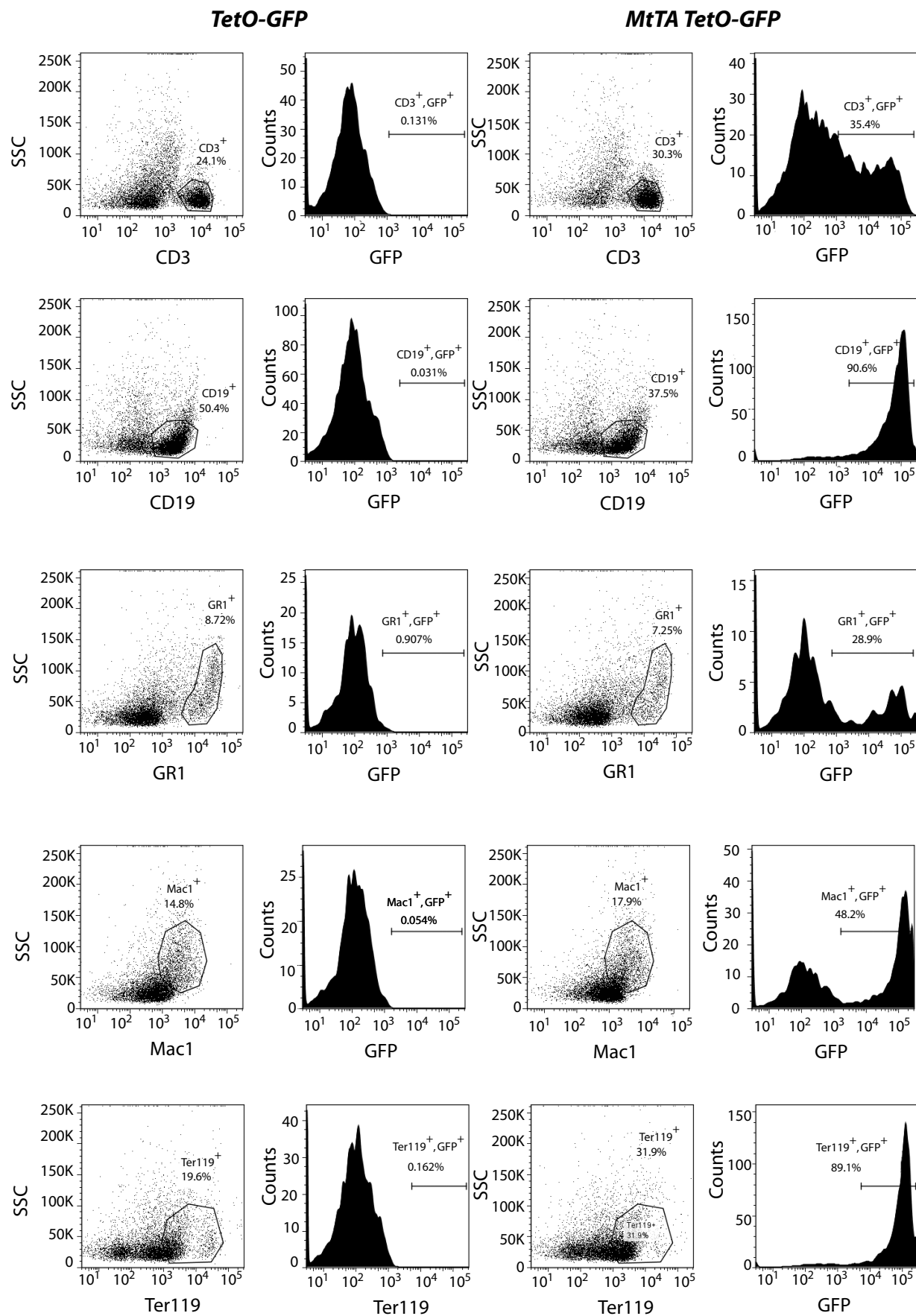

Supplement: Figure S2 — Representative flow cytometric blots showing the presence of GFP in differentiated hematopoietic lineages of MMTV-tTA TetO-GFP double transgenic mice and their TetO-GFP single transgenic controls. These blots correspond to the bar graph shown in Fig. 2B. (PDF) [file pone.0060902.s002.pdf]

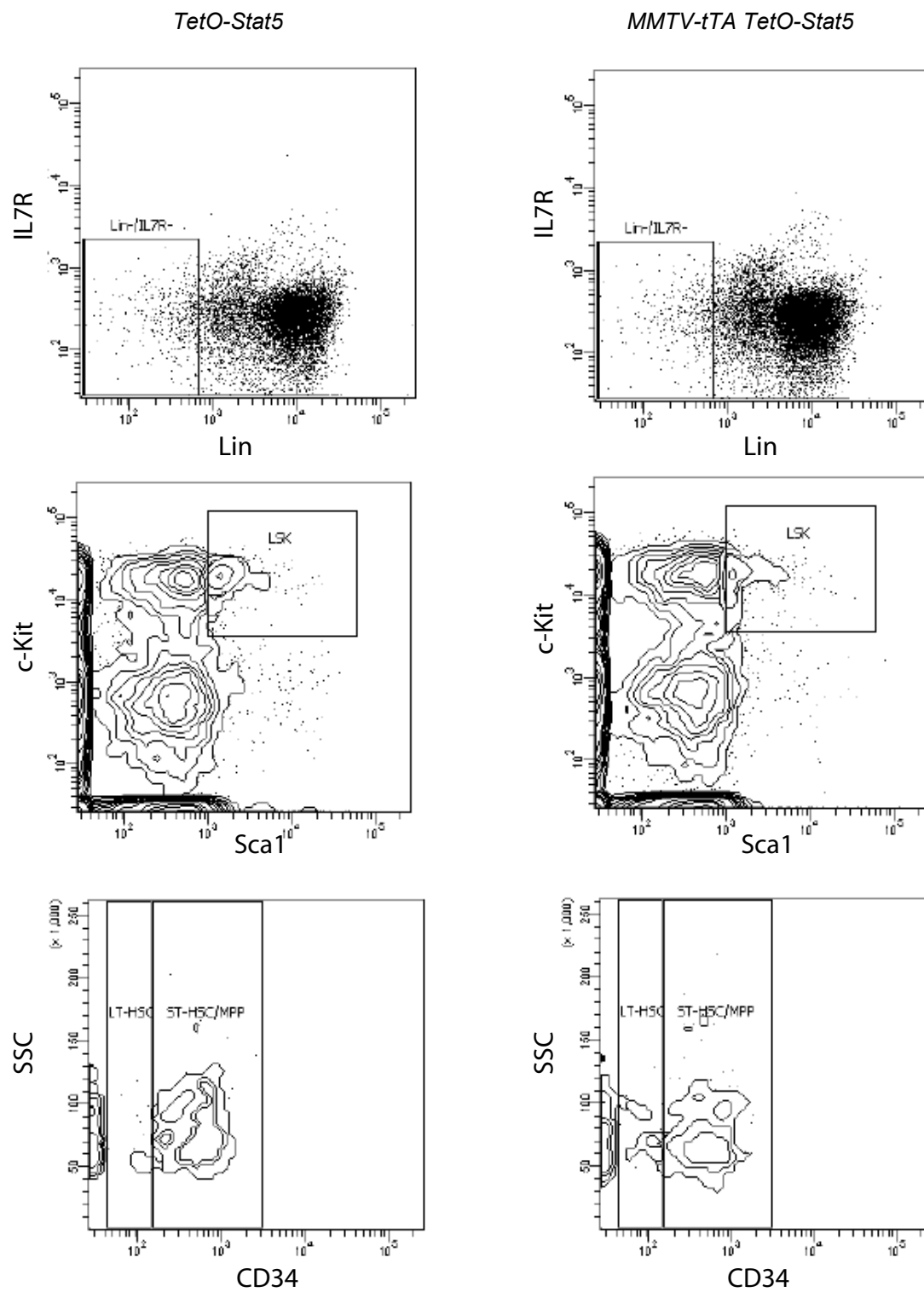

Supplement: Figure S3 — Representative flow cytometric blots of the relative numbers of long-term and short-term (LT-HSC, ST-HSC) hematopoietic stem cells in the bone marrow of MMTV-tTA TetO-Stat5 double transgenic mice and their TetO-Stat5 single transgenic controls. These blots correspond to the quantitative analysis shown in Fig. 3A. (PDF) [file pone.0060902.s003.pdf]

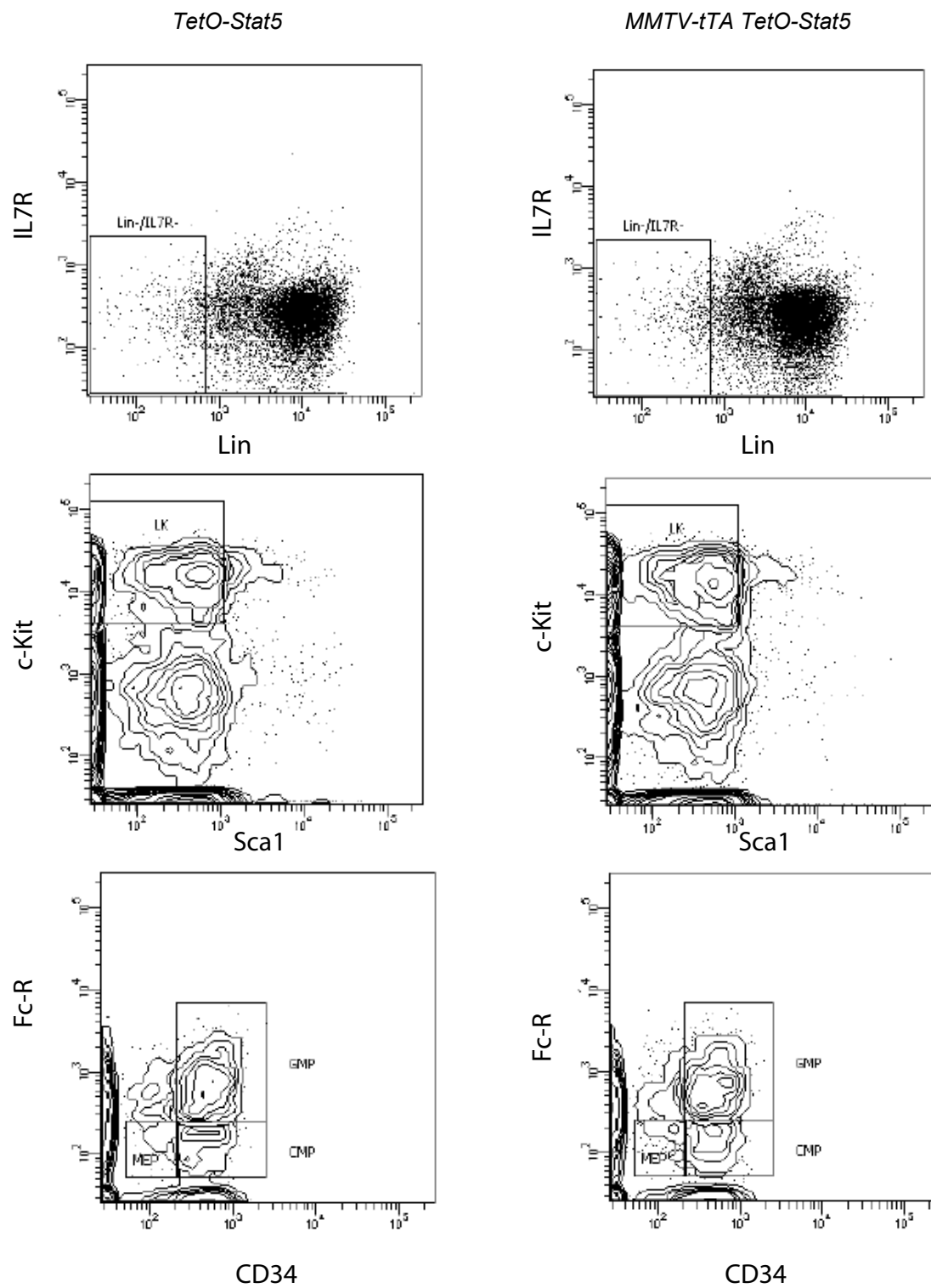

Supplement: Figure S4 — Representative flow cytometric blots of common myeloid (CMP), granulocyte-macrophage (GMP), and megakaryocyte-erythrocyte (MEP) progenitors in the bone marrow of MMTV-tTA TetO-Stat5 double transgenic mice and their TetO-Stat5 single transgenic controls. These blots correspond to the quantitative analysis shown in Fig. 3B. (PDF) [file pone.0060902.s004.pdf]

Figure S5

Lin and Schmidt et al.

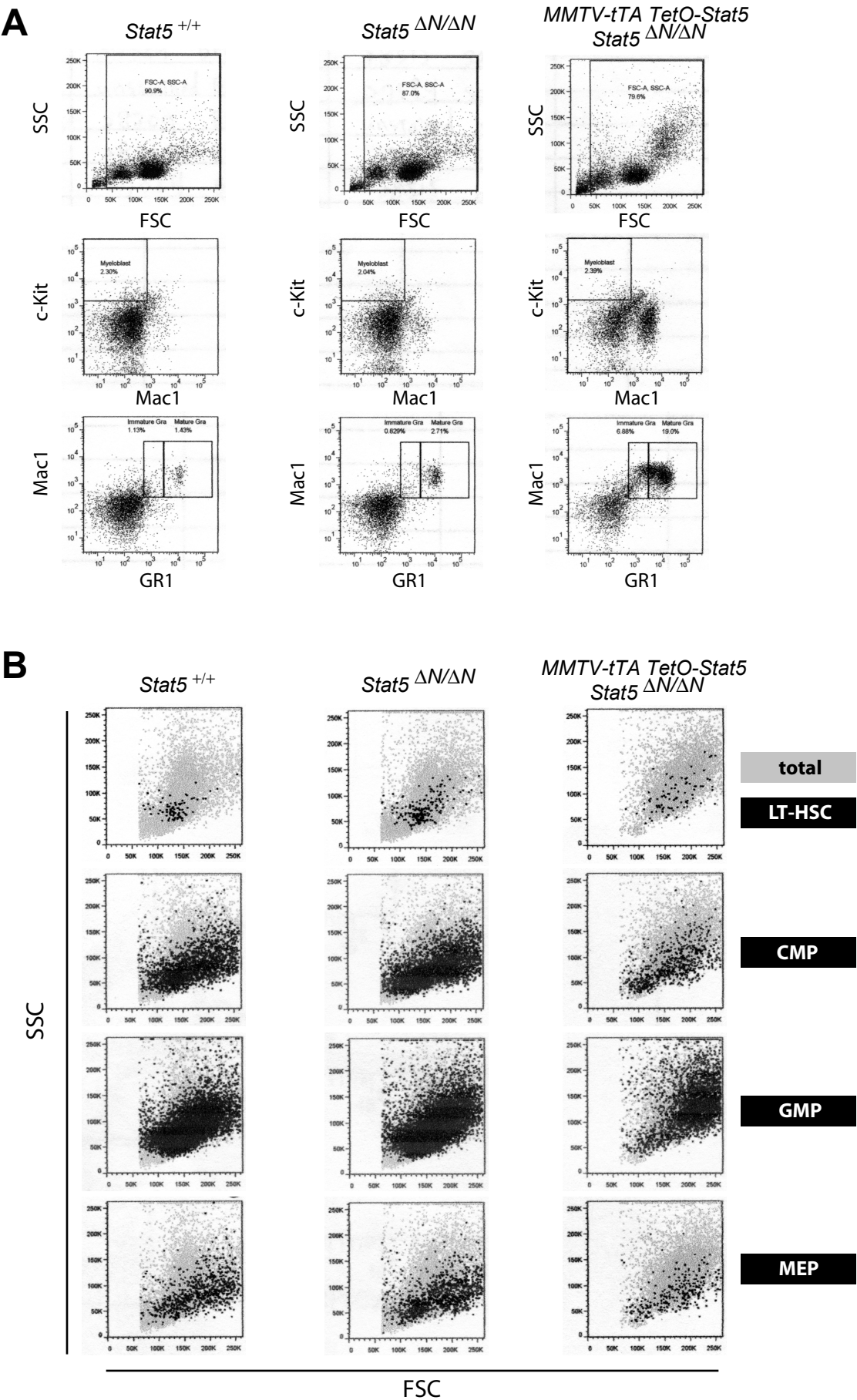

Supplement: Figure S5 — Representative flow cytometric blots of the contribution of myeloblasts and immature and mature granulocytes among the total number of splenocytes (A) and numbers of long-term hematopoietic stem cells (LT-HSC) as well as common myeloid (CMP), granulocyte-macrophage (GMP), and megakaryocyte-erythrocyte (MEP) progenitors (B) in the bone marrows of diseased MMTV-tTA TetO-Stat5 Stat5 Δ N/ΔN mice and their controls. The blots shown in panel A correspond to the bar graph illustrated in the left panel of Fig 5A. The blots from the backgating shown in panel B correspond to the bar graph shown in the right panel of Fig 5A. (PDF) [file pone.0060902.s005.pdf]

**Figure S6**

Lin and Schmidt et al.

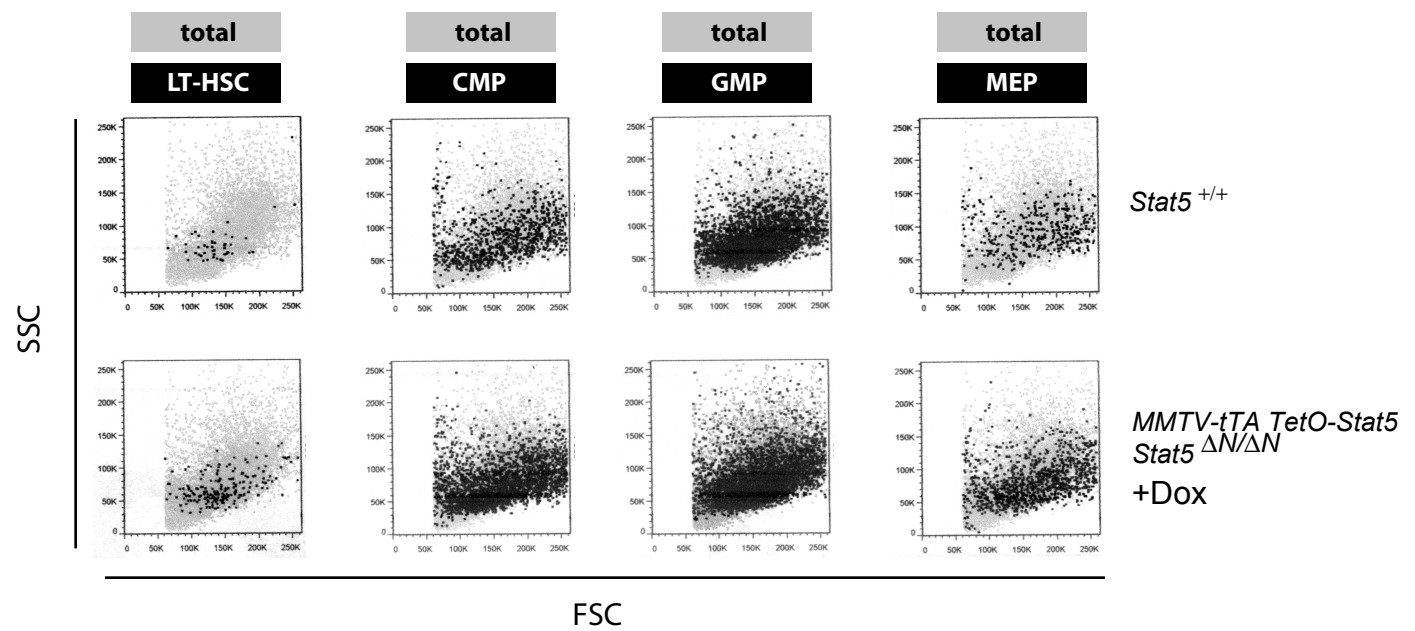

Supplement: Figure S6 — Representative flow cytometric blots of long-term hematopoietic stem cells (LT-HSC) as well as common myeloid (CMP), granulocyte-macrophage (GMP), and megakaryocyte-erythrocyte (MEP) progenitors in the bone marrows of a wildtype control and a diseased MMTV-tTA TetO-Stat5 Stat5 Δ N/ΔN mouse following Dox administration. These backgated blots against the total number of bone marrow cells correspond to the bar graph shown in Fig 5C. (PDF) [file pone.0060902.s006.pdf]
